# An undernutrition screening score for dogs with protein‐losing enteropathy: A prospective multicenter study

**DOI:** 10.1111/jvim.16794

**Published:** 2023-07-21

**Authors:** Florence E. Wootton, Christopher S. F. K. Hoey, Glynn Woods, Silke Salavati Schmitz, Jenny Reeve, Jennifer Larsen, Aarti Kathrani

**Affiliations:** ^1^ Department of Clinical Science and Services Royal Veterinary College Hatfield UK; ^2^ Bristol Veterinary School University of Bristol Bristol UK; ^3^ University of Edinburgh, The Royal (Dick) School of Veterinary Studies, Hospital for Small Animals Easter Bush UK; ^4^ Department of Molecular Biosciences, School of Veterinary Medicine University of California Davis Davis California USA

**Keywords:** canine, diarrhea, gastrointestinal, malnutrition

## Abstract

**Background:**

The impact of undernutrition in dogs with protein‐losing enteropathy (PLE) caused by inflammatory enteritis, intestinal lymphangiectasia, or both and which variables are most predictive of outcome are unknown.

**Objectives:**

Develop an undernutrition screening score (USS) for use at the time of diagnosis of PLE in dogs, which is predictive of outcome.

**Animals:**

Fifty‐seven dogs with PLE prospectively recruited from 3 referral hospitals in the United Kingdom.

**Methods:**

An USS based on the presence and severity of 5 variables: appetite, weight loss, and body, muscle, and coat condition and scored out of 15, with higher scores reflecting worse undernutrition, was calculated at the time of diagnosis. Follow‐up information was obtained for at least 6 months.

**Results:**

Dogs that failed to achieve clinical remission within 6 months had higher USS at diagnosis compared with dogs that achieved remission (median, 7.5; range, 2‐14 and median, 5; range, 0‐14, respectively). The USS at diagnosis gave an area under the receiver operating characteristic curve (AUC) of 0.656 for predicting nonclinical remission within 6 months, whereas a score consisting of just epaxial muscle loss and coat condition resulted in a larger AUC of 0.728.

**Conclusions and Clinical Importance:**

Of the 5 variables assessed in the USS, a combination of epaxial muscle loss and coat condition was most predictive of not achieving clinical remission within 6 months in dogs with PLE. Additional studies will help determine the effect of changes in USS and the 5 associated variables after diagnosis on outcome variables in these dogs.

**Abbreviations**
BCS: body condition score
BMI: body mass index
CCECAI: canine chronic enteropathy clinical activity index
GI: gastrointestinal
IBD: inflammatory bowel disease
USS: undernutrition screening score

## 1 INTRODUCTION

Undernutrition screening tools are used in human hospitals to identify those at risk of nutritional deficiency, which may then result in referral for nutritional assessment and intervention.^1^ The 3 main accepted findings required to make a diagnosis of undernutrition in humans are weight loss, decreased body mass index, and decreased fat‐free mass (primarily muscle mass).^2^, ^3^ Undernutrition is present in up to 70% of humans with active inflammatory bowel disease (IBD) and 38% of those in remission.^2^ Humans with IBD who are identified to be undernourished have increased number and duration of hospitalizations, disease flares, complications, need for surgery, and death.^2^

Nutritional assessment as a formal process in small animal veterinary medicine has been promoted for over a decade.^4^ It includes collection and evaluation of both categorical and noncategorical data, which is less discrete and typically requires additional interpretation, followed by integration of all of this information by the practitioner to provide recommendations. Given the impact of chronic enteropathy on food intake and nutrient absorption in veterinary patients, an additional and more straightforward evaluation with a quantitative score is warranted to help guide treatment and inform prognosis.

Dogs with protein‐losing enteropathy (PLE) caused by inflammatory enteritis, intestinal lymphangiectasia, or both have a guarded prognosis, and death occurs as a result of the disease in 54.2% of cases.^5^ The histopathologic findings of dogs with PLE are different from those of humans with IBD. However, many factors associated with the clinical presentation and pathogenesis, which put people with IBD at risk of undernutrition, such as decreased dietary intake and malabsorption, also are seen in dogs with PLE.^6^, ^7^ Dogs with PLE often have chronic and relapsing gastrointestinal (GI) signs such as vomiting, small and large bowel diarrhea, abdominal discomfort, excessive borborygmi, weight loss, and altered appetite.^6^, ^8^ It is therefore likely that the high prevalence of undernutrition and its consequences seen in humans with IBD are present in dogs with PLE. Nutritional assessment is not always taken into consideration by clinicians at the time of diagnosis of PLE. Therefore, a specific and straightforward nutritional screening score for these patients could improve recognition of undernutrition.

Our primary aim was to establish a clinical undernutrition screening score (USS) that was quantitative, easy to perform, and with a low level of interobserver variation to help determine the prevalence of undernutrition in dogs with PLE at diagnosis. Our secondary aim was to identify if the USS, scored at diagnosis, was associated with outcome in this cohort of dogs. Our tertiary aim was to determine which combination of variables used for the USS at diagnosis was most predictive of outcome. Unfortunately, the assessment of caloric intake and serial USS after diagnosis were beyond the scope of our study. Our main hypothesis was that undernutrition would be common in a population of dogs with PLE. Our secondary hypothesis was that undernutrition would have a significant negative impact on outcome. Our tertiary hypothesis was that an USS consisting of all 5 variables would be most predictive of outcome.

## 2 MATERIALS AND METHODS

### 2.1 Study design

Our study was a prospective multicenter observational study. Dogs were recruited from 3 referral teaching hospitals in the United Kingdom between July 2019 and June 2021. Dogs suspected to have a PLE with hypoalbuminemia (<2.8 g/dL) in association with GI signs were prospectively recruited at the time of obtaining GI biopsy specimens.

Cases of PLE were included if they had complete diagnostic investigations to rule out other causes of GI signs and hypoalbuminemia, along with a histopathologic diagnosis of inflammatory enteritis, intestinal lymphangiectasia, or both, regardless of duration of clinical signs. Diagnostic investigations were considered complete if they included the following: CBC, serum biochemistry, serum vitamin B_12_ concentration, abdominal imaging, and fecal parasitology with or without a 5‐day course of fenbendazole. Serum basal cortisol concentration was measured in those dogs that had changes on their CBC suggestive of hypoadrenocorticism, such as lymphocytosis or eosinophilia. An adrenocorticotropic hormone (ACTH) stimulation test was performed on those dogs with basal cortisol concentration <2 μg/dL (<55 nmol/L).^9^ Basal serum cortisol concentration was not obtained in dogs that had recently received exogenous glucocorticoids. Dogs without urinalysis were included if they had pan‐hypoproteinemia and low or low‐normal serum cholesterol concentrations, and histopathologic findings compatible with a diagnosis of inflammatory enteritis, intestinal lymphangiectasia, or both. All histopathologic assessments were performed by a board‐certified veterinary pathologist or a specialist‐in‐training under direct supervision of a board‐certified pathologist.

Cases with incomplete diagnostic investigations and those with a histopathologic diagnosis of neoplasia were excluded. Dogs that died after diagnosis, before discharge, were included in the study. Dogs with substantial proteinuria (urine protein:creatinine ratio >0.5) were excluded because of the effect it could have on serum albumin concentrations. If urinalysis was not performed, dogs were excluded if they had serum biochemical changes consistent with a protein‐losing nephropathy (PLN), such as hypercholesterolemia with normal or increased serum globulin concentration.

Medical records were reviewed for each dog and the signalment, clinical history, duration of clinical signs, and treatment administered before referral were recorded. Baseline diagnostic tests were recorded when performed. Because the reference interval for serum vitamin B_12_ concentration was different at the 3 institutions, the interval from the institution that recruited the largest number of cases was used to categorize the concentrations for all dogs. Therefore, dogs with a serum vitamin B_12_ concentration >200 ng/L were categorized as within the reference interval and those ≤200 ng/L as lower than the reference interval. The results of additional diagnostic tests such as canine pancreatic lipase, trypsin‐like immunoreactivity, and fecal culture were recorded when performed.

### 2.2 Development of an USS

**FIGURE 1:**
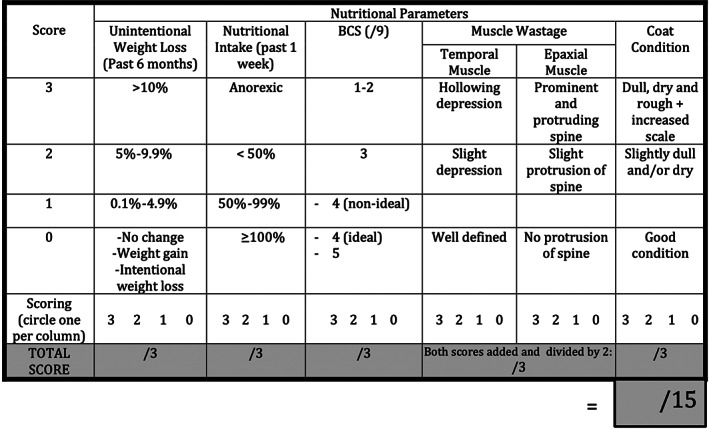
The undernutrition screening score used for this study. The score consisted of 5 parameters: (1) Unintentional weight loss in the past 6 months, (2) Nutritional intake, as perceived by the owner in the last 7 days before presentation, (3) Body condition score assessed on a 9‐point scale, (4) Muscle condition assessed using the epaxial and temporal muscles, and (5) Coat condition. As two muscle groups were used in the assessment of muscle condition, the score for both was added and halved when calculating the total score (shaded row, added after data collection), to ensure equal weighting of all 5 parameters. The maximum possible total score was 15.

**FIGURE 2:**
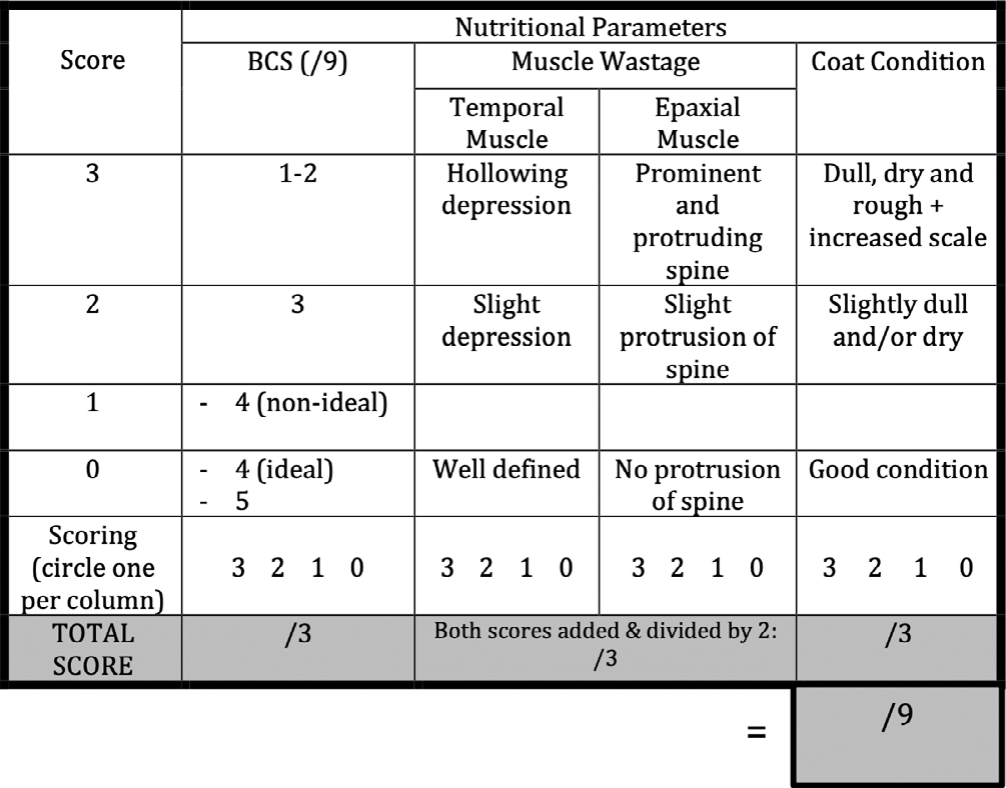
The abbreviated undernutrition screening score used to assess the interobserver variation of the physical parameters used in the full score. The physical parameters consisted of: body condition score assessed on a 9‐point scale, muscle condition assessed using the epaxial and temporal muscles, and coat condition. As two muscle groups were used in the assessment of muscle condition, the score for both was added and halved when calculating the total score (shaded row, added after data collection), to be in line with the full scoring (Figure 1). The maximum possible total score for the abbreviated scoring was 9.

Undernutrition is not well defined in the small animal veterinary population, but 1 study defined it as a change in body condition score (BCS) and body weight.^10^ Therefore, the human medical literature was used to help develop an USS for our study.^1^, ^2^, ^3^ Because our aim was to develop a system that was easy to use, we focused on the 3‐Minute Nutrition Screening used in human medicine.^11^ In addition to the 3 variables used in the 3‐Minute Nutrition Screening (weight loss, intake, and muscle wasting), we also incorporated BCS and coat condition in our score. Therefore, our score consisted of the following 5 nutritional variables (Figure 1):

1. *Unintentional weight loss in the previous 6 months*
  This variable included 4 categories and was scored out of 3, with 0: no change, weight gain, or intentional loss, 1: 0.1% to 4.9% body weight loss, 2: 5% to 9.9% body weight loss, and 3: ≥10% body weight loss.
2. *Nutritional intake over the previous 7 days*
  This variable included 4 categories and was scored out of 3, with 0: ≥100% of normal intake as perceived by the owner, 1: 50% to 99% of normal intake as perceived by the owner, 2: <50% of normal intake as perceived by the owner, and 3: 0% intake (anorexic).
3. Body condition score
  BCS on a 9‐point scale^12^ was divided into 4 categories, with 0:  5/9 or 4/9 with the clinician assessing the dog as ideal, 1:  4/9 and not ideal (where 5 would instead be considered ideal for that dog), 2:  3/9, and 3 = 1 to 2/9. For those dogs with a BCS >5, a score of 0 was given.
4. *Muscle condition*
  Muscle condition was assessed using the epaxial and temporal muscles and categorized as either no loss (score of 0), mild loss (score of 2), or severe loss (score of 3). Because 2 muscle groups were used in the assessment of muscle condition, the score for both was added and halved when calculating the total score (shaded row in Figures 1 and 2, added after data collection) to ensure equal weighting of all 5 variables.
5. *Coat condition*
  Coat condition was assessed and categorized as either good condition (score of 0), slightly dry or dull or both (score of 2) or dull, dry and rough with increased scale (score of 3).
  The total USS for each case was calculated once at histopathologic diagnosis, with a maximum possible score of 15. The USS was performed by a small animal internal medicine specialist or a specialist in training for all cases. The total USS scores were subjectively subcategorized as not clinically relevant for 0 to <4, mild for ≥4 to <8, moderate for ≥8 to <12, and severe for ≥12.
  The abbreviated USS was performed to assess the interobserver variation of the subjective physical measurements in the USS. An abbreviated USS (Figure 2), which focused on just the subjective physical measurements of BCS, muscle, and coat condition, was performed by a second observer blinded to the score of the first assessor in a subset of cases. The maximum possible score of the abbreviated USS was 9. The abbreviated USS results were subjectively subcategorized into the following categories depending on the total score: not clinically relevant to mild for 0 to <4, moderate for 4 to <7, and severe for 7 to 9. The second assessment was performed in person by either a small animal internal medicine specialist or a specialist or intern in training.

### 2.3 Treatment

As ours was an observational study, treatment prescribed was at the discretion of the attending veterinarian. Treatment prescribed at diagnosis was recorded and cases were categorized into the following subgroups: diet alone, diet with antibiotics, diet with glucocorticoids, diet with glucocorticoids and a second immunosuppressive agent or diet with antibiotics and glucocorticoids. Additional treatment such as cobalamin supplementation, antiplatelet medication, and symptomatic treatment was recorded when administered. Placement of an enteral feeding tube also was recorded when performed.

### 2.4 Follow‐up

All cases were followed for a minimum of 6 months after histopathologic diagnosis or until the time of death or euthanasia if within 6 months. Follow‐up information was obtained from re‐examination visits at the same referral teaching hospital or from referring veterinarians or from both sources. Response to treatment prescribed at diagnosis was considered successful based on information from owner and referring veterinarian follow‐up, and was defined as resolution of clinical signs without the need for a change or additional treatment from that prescribed at diagnosis or when glucocorticoids could be tapered without relapse. Cases were considered to have failed to respond to treatment prescribed at diagnosis if a change in treatment, additional treatment, or euthanasia was required because of persistent GI signs or serum biochemical abnormalities. Clinical remission was defined as resolution of all GI signs and serum biochemical remission was defined as normalization of serum albumin concentration by the end of the 6‐month follow‐up period. Cases that achieved clinical and biochemical remission included both those in which treatment prescribed at diagnosis was successful and those that initially failed to respond to treatment prescribed at diagnosis but then achieved remission with a subsequent change or additional treatment. The assay used to measure serum albumin concentrations postdiagnosis was variable, because most dogs had repeat laboratory work performed at their primary care practice with different reference intervals. Therefore, to ensure correct classification of cases, the serum albumin concentration was divided by the lower limit of the reference interval and a result of ≥1 was considered to indicate biochemical remission.

Survival analysis was based on survival to 6 months after histopathologic diagnosis. If dogs died or were euthanized for reasons unrelated to GI disease, the cause of death was established and recorded. Dogs that died or were euthanized for reasons unrelated to PLE, or if the cause of death was not established, were removed from survival analysis.

### 2.5 Statistical analysis

Statistical analysis was performed using statistical package for the social sciences (SPSS) v26 (IBM Corp). Normality was assessed for continuous variables using the Shapiro‐Wilk test. Results were reported as mean ± SD if normally distributed and as medians with range if not normally distributed.

The Mann‐Whitney *U* test was used to compare the USS scores with binary outcome variables. The following binary outcome variables were analyzed for significance: whether or not dogs were alive at 6 months, had responded to treatment prescribed at diagnosis, achieved clinical remission, achieved serum biochemical remission, and had been hospitalized for ≥5 days. The correlation between USS and duration of clinical signs was assessed using Spearman's rank correlation coefficient. A correlation coefficient (*r*_s_) of ±0.9 to 1.0 was interpreted as very high, ±0.90 to 0.70 as high, ±0.70 to 0.50 as moderate, ±0.50 to 0.30 as low, and ±0.30 to 0.00 as negligible.^13^ Significance was defined as *P* < .05.

The interobserver variation for the USS was assessed using Cohen's kappa. Survival analysis was performed using Kaplan‐Meier curves for the USS. Cases that were not clinically relevant or that were mild were grouped together for the USS in survival analysis because of the small case numbers. Cases were right censored if the dogs were alive at the time of last follow‐up.

Receiver operating characteristic curves were used to determine which individual variables of the USS were most predictive of death within 6 months or not achieving clinical remission within 6 months, utilizing 2 separate models. The scores of individual variables that resulted in an area under the curve (AUC) of >0.6 were combined to determine the best overall combination of USS variables at predicting outcome. An AUC of >0.9 was interpreted as excellent, 0.8 to 0.89 as good, 0.7 to 0.79 as fair, 0.6 to 0.69 as poor, and 0.5 to 0.59 as failure.^14^

## 3 RESULTS

### 3.1 Study population

Fifty‐seven dogs with PLE were included in the study: 13 intact males, 20 neutered males, 5 intact females and 19 spayed females. Thirty‐five dogs had diarrhea, 1 dog had vomiting alone and 21 had both diarrhea and vomiting at the time of presentation. The median age was 6.8 years (range, 0.4‐11.9). There were 50 purebred dogs (88%) and 7 crossbreeds (12%). The most common breeds were Cavalier King Charles spaniel (n = 4), Staffordshire bull terrier (n = 4), border collie (n = 4), miniature schnauzer (n = 3), and pug (n = 3).

The median duration of clinical signs before presentation was 90 days (range, 14‐1260). The median body weight was 10.3 kg (range, 1.9‐41.6). The median duration of hospitalization was 5 days (range, 1‐14).

### 3.2 Treatment at the time of referral and glucocorticoid administration before referral

Four dogs were receiving prednisolone at the time of referral: 1.85 mg/kg/day for 3 weeks (n = 1), 3 mg/kg/day for 3 weeks (n = 1), 1 mg/kg/day for 1 week (n = 1), 1.7 mg/kg/day starting dose tapered over 4 months to 1.5 mg/kg/day with chlorambucil at 4 mg/m^2^ every other day for 2 months (n = 1). One dog had received a single dose of dexamethasone SC the day before presentation. No other dogs had received or were receiving glucocorticoids at the time of referral.

One dog was receiving Grapiprant for osteoarthritis and 1 was receiving oclacitinib for atopic dermatitis.

### 3.3 Biochemical data and intestinal histopathology

The mean serum albumin concentration at presentation was 1.8 ± 0.5 g/dL (reference interval, 2.8‐3.8 g/dL). All dogs included in the study had intestinal biopsies performed. Detailed serum biochemical data and intestinal histopathology results are included in the Data S1.

### 3.4 Undernutrition screening score

Results for each variable of the USS are summarized in Table 1. The median total score was 6 (range, 0‐14). Only 13/57 cases (23%) had nonclinically relevant USS of <4 with 44/57 (77%) cases having at least mild undernutrition with USS ≥4. Overall, unintentional weight loss was identified in 46/57 (81%) of dogs and appetite was decreased in 31/57 (54%). Low BCS (<ideal of 4‐5/9) was present in 37/57 (65%) cases. Temporal muscle loss was evident in 38/57 (67%) and epaxial muscle loss in 39/57 (68%) cases. Coat condition was abnormal in 32/57 (56%) cases.

**TABLE 1:** Results for each variable of the undernutrition screening score for 57 dogs with protein‐losing enteropathy calculated at the time of obtaining intestinal biopsy specimens.

USS variable	Category	Number of cases (%)
Unintentional weight loss	No change, weight gain, or intentional weight loss	11 (19%)
	Mild (0.1%‐4.9%)	17 (30%)
	Moderate (5%‐9.9%)	9 (16%)
	Severe (≥10%)	20 (35%)
Nutritional intake	Normal or increased (≥100%)	26 (46%)
	Mildly decreased (50%‐99%)	9 (16%)
	Moderately decreased (<50%)	14 (24%)
	Anorexic (nil intake)	8 (14%)
Body condition score (/9)	Normal (4 [ideal for dog as assessed by clinician] or 5) or overweight	20 (35%)
	Mildly underconditioned (4 [non‐ideal for dog as assessed by clinician, where instead 5 would be considered ideal])	10 (18%)
	Moderately underconditioned (3)	16 (28%)
	Severely underconditioned (1, 2)	11 (19%)
Temporal muscle condition	Well defined	19 (33%)
	Slight depression	31 (55%)
	Hollowing	7 (12%)
Epaxial muscle condition	No protrusion of spine	18 (32%)
	Slight protrusion of spine	24 (42%)
	Prominent and protruding spine	15 (26%)
Coat condition	Good condition	25 (44%)
	Slightly dry and/or dull	29 (51%)
	Dull, dry, and rough with increased scale	3 (5%)

Abbreviation: USS, undernutrition screening score.

### 3.5 Treatment at diagnosis

Treatment administered at diagnosis of PLE was variable and consisted of diet alone (n = 12), diet with antibiotics (n = 4), diet with glucocorticoids (n = 36), diet with glucocorticoids and a second immunosuppressive agent (n = 2), or diet with antibiotics and glucocorticoids (n = 3). An enteral feeding tube was placed in 13 dogs (23%). Information regarding additional treatment administered at diagnosis is included in the Data S1.

### 3.6 Outcome

Thirty‐seven dogs (65%) were alive 6 months after histopathologic diagnosis. Sixteen dogs (28%) died or were euthanized as a result of PLE within 6 months of histopathologic diagnosis. Three dogs died or were euthanized for reasons unrelated to the PLE (traumatic brain injury, ethylene glycol toxicity, and ocular lymphoma) and 1 dog died suddenly at home 5 months after diagnosis with no cause established.

Thirty‐four dogs (60%) failed to respond to treatment prescribed at diagnosis with the remaining 23 dogs (40%) responding to initial treatment. Twenty‐eight dogs (49%) achieved clinical remission within 6 months of diagnosis. Follow‐up serum biochemistry was performed in 46 dogs, 23 dogs (40%) achieved serum biochemical remission within 6 months, with the remaining 23 dogs (40%) failing to achieve remission. Follow‐up laboratory testing was not performed in 11 dogs (19%).

Duration of clinical signs and USS were not correlated (*P* = .76, *r*_s_ = 0.041). All results for USS are summarized in Table 2. Dogs that were hospitalized for ≥5 days had higher USS at histopathologic diagnosis. Dogs that failed to respond to treatment prescribed at diagnosis had higher USS. Dogs that failed to achieve clinical remission within 6 months of histopathologic diagnosis had higher USS. However, dogs that failed to achieve serum biochemical remission within 6 months of histopathologic diagnosis had USS not significantly different from those that achieved serum biochemical remission. Furthermore, dogs that died or were euthanized because of PLE within 6 months of histopathologic diagnosis had USS that was not significantly different from those that survived.

**TABLE 2:** The total undernutrition screening score (USS) in positive and negative outcome groups.

Outcome variable (positive outcome and negative outcome)	Median USS of positive outcome (range)	Median USS of negative outcome (range)	*P* value (MW)
Duration of hospitalization (<5 days or ≥5 days)	4.75 (0‐12)	9 (1‐14)	**.002**
Response to treatment prescribed at diagnosis (controlled or uncontrolled)	5 (0‐13)	8 (1‐14)	**.007**
Clinical remission (achieved or failure)	5 (0‐14)	7.5 (2‐14)	**.04**
Biochemical remission (achieved or failure)	5 (0‐14)	7 (1‐13)	.58
Survival to 6 months (alive or dead)	6 (0‐14)	8.75 (1‐14)	.28

*Note*: The column “*P* value (MW)” represents the *P* value from the Mann‐Whitney *U* test when comparing the USS scores from the positive and negative outcome groups for the listed outcome variables below. Statistically significant results are highlighted in bold.

Abbreviations: MW, Mann‐Whitney *U* test; USS, undernutrition screening score.

No significant difference was found in survival distributions between subgroups of insignificant and mild, moderate, and severe USS (*P* = .79). Because fewer than 50% of dogs died during our follow‐up period for most individual subgroups, median survival times could not be determined.

An abbreviated USS consisting of the 3 subjective physical variables of BCS, muscle condition, and coat condition was performed in 26 dogs (46%). When comparing results for the abbreviated USS between the 2 independent assessors, the agreement was substantial with a Cohen's kappa *k* = 0.747 (*P* < .001).^15^

Receiver operating characteristic (ROC) curves showed USS was not predictive of death within 6 months of diagnosis (AUC, 0.604; *P* = .2). However, the combined score of epaxial muscle loss and coat condition was most predictive of death within 6 months (AUC, 0.687; *P* = .02; Table 3). Similarly, for the outcome of not achieving clinical remission within 6 months, ROC curves showed USS was not predictive (AUC, 0.656; *P* = .05). However, the combined score of epaxial muscle loss and coat condition was most predictive of not achieving clinical remission within 6 months (AUC, 0.728; *P* = .004; Table 4). For the combined score of epaxial muscle loss and coat condition, a cutoff of 2.5 was the best predictor of death within 6 months, with a sensitivity of 66.7% and specificity of 73.9%, and the best predictor of not achieving clinical remission within 6 months, with a sensitivity of 71.4% and specificity of 76.0% (Tables 3 and 4, respectively).

**TABLE 3:** Receiver operating characteristic curve results for predicting death within 6 months of diagnosis of protein‐losing enteropathy.

Variable	AUC	SE AUC	*P*‐value	95% CI
USS	0.604	0.079	.20	0.449‐0.759
Intake	0.572	0.082	.37	0.412‐0.733
Weight loss	0.593	0.079	.25	0.439‐0.748
BCS	0.457	0.080	.60	0.301‐0.614
Temporal muscle loss	0.541	0.081	.62	0.382‐0.699
Epaxial muscle loss	0.615	0.080	.15	0.459‐0.771
Combined muscle loss score	0.541	0.080	.62	0.385‐0.698
Coat condition	0.614	0.078	.16	0.462‐0.766
Epaxial condition + coat condition	0.687	0.076	.02^a^	0.537‐0.837

Abbreviations: AUC, area under the curve; BCS, body condition score; CI, confidence interval; SE, standard error; USS, undernutrition screening score.

^a^ Best cut‐off, sensitivity, and specificity for epaxial condition + coat condition: 2.5, 66.7%, and 73.9%, respectively.

**TABLE 4:** Receiver operating characteristic curve results for predicting nonclinical remission within 6 months of diagnosis of protein‐losing enteropathy.

Variable	AUC	SE AUC	*P* value	95% CI
USS	0.656	0.076	.05	0.508‐0.805
Intake	0.594	0.080	.24	0.437‐0.750
Weight loss	0.619	0.078	.14	0.466‐0.773
BCS	0.508	0.080	.92	0.350‐0.666
Temporal muscle loss	0.557	0.080	.48	0.401‐0.714
Epaxial muscle loss	0.639	0.077	.08	0.487‐0.790
Combined muscle loss score	0.521	0.080	.80	0.364‐0.677
Coat condition	0.654	0.075	.06	0.506‐0.801
Epaxial condition + coat condition + weight loss	0.712	0.072	0.01^a^	0.571‐0.853
Epaxial condition + coat condition	0.728	0.073	0.004^b^	0.586‐0.870

Abbreviations: AUC, area under the curve; BCS, body condition score; CI, confidence interval; SE, standard error; USS, undernutrition screening score.

^a^ Best cut‐off, sensitivity, and specificity for epaxial condition + coat condition + weight loss: 4.5, 64.3%, and 68.0%, respectively.

^b^ Best cut‐off, sensitivity, and specificity for epaxial condition + coat condition: 2.5, 71.4%, and 76.0%, respectively.

## 4 DISCUSSION

A high prevalence of undernutrition was found based on the results of the USS, with 77% of dogs in our study having at least mild undernutrition at the time of diagnosis. In addition, higher USS scores were associated with increased duration of hospitalization, failure to respond to treatment prescribed at diagnosis, and failure to achieve clinical remission within 6 months of histopathologic diagnosis. The combination of epaxial muscle loss and coat condition was found to be the best predictor of death and not achieving clinical remission within 6 months of diagnosis. These findings suggest that the nutritional status of dogs with PLE at diagnosis may have a clinically relevant effect on outcome variables and highlights the importance of assessing at least epaxial muscle and coat condition in affected dogs.

The high prevalence of undernutrition identified in our study highlights the need for increased awareness of this problem in dogs with PLE among clinicians. Increased awareness could result in earlier recognition, diagnosis, and intervention in affected dogs. A previous, retrospective study in dogs identified improved outcome in dogs with PLE that received targeted nutritional support as part of their treatment.^16^ It is likely that the positive effects were in part a result of reversing the physiological effects of undernutrition. Additional prospective studies should be performed to identify if the USS could help determine when assisted enteral feeding should be considered for dogs with PLE and if such treatment could help improve outcome variables. Despite 22/57 (38%) affected dogs having moderately to severely decreased appetite at the time of intestinal biopsy, feeding tubes only were placed in 13/57 (23%) dogs. The cause for this discrepancy was not established and could be related to several factors including owner financial limitations or reluctance to place a feeding tube, patient demeanor, and clinician lack of awareness of the potential benefits of tube placement and assisted enteral feeding.

No statistically significant difference was found in USS between nonsurvivors and survivors. This observation could be related to small sample size and relatively low fatality rate in this cohort or the duration of follow‐up (type II error). However, this finding also could indicate that undernutrition does not impact overall survival in dogs with PLE, in contrast to humans with IBD^2^ or that different variables or combinations of variables associated with undernutrition are needed to predict survivors versus nonsurvivors.

The integumentary system is an important variable that is impacted by undernutrition and is routinely assessed when determining nutritional status. Nutritional deficiencies often manifest themselves as poor coat condition and nutrients such as zinc and linoleic acid have been demonstrated to significantly improve coat condition in dogs.^17^ Deficiencies of vitamins, polyunsaturated fatty acids, and zinc are uncommon in dogs because of the common use of complete commercial diets.^18^ It is likely that dogs with PLE develop nutritional deficiencies that impact coat condition because of decreased dietary intake, decreased absorption, or increased intestinal losses. Our results further highlight the need to ensure that assessment of coat condition is taken into consideration during nutritional assessment of dogs with PLE.

Three previous studies have assessed the effect of BCS on outcome in dogs with PLE but none of these studies assessed muscle condition.^6^, ^8^, ^16^ Ours is the first study to report muscle condition in PLE, which is more relevant to nutritional status and is consistent with nutritional screening tools used in humans with IBD, which encompass various physical variables of nutritional status in addition to body mass index.^19^ Two muscle groups were used to assess muscle condition in our study, based on the 3‐Minute Nutrition Screening which uses 2 groups, each with their own score.^11^ In addition, because muscle loss can be localized or diffuse, we assessed 2 areas. We decided to use temporalis and epaxial muscle loss, because we perceived these to be less likely affected by orthopedic disease. The World Small Animal Veterinary Association recommends a 3‐point system for mild, moderate, and severe loss. However, we chose to categorize muscle loss as either no loss (score of 0), mild loss (score of 2), or severe loss (score of 3) for 2 reasons. Firstly, we aimed to minimize subjectivity and improve interobserver agreement, because a previous study suggested that mild and moderate categories of muscle loss be combined in dogs to improve agreement.^20^ In addition, this grading of mild and severe loss is the same one as in the 3‐Minute Nutrition Screening, which was shown to be valid and accurate for determining malnutrition in humans. Our results in particular highlighted the importance of assessing epaxial muscle loss when performing nutritional assessment in dogs with PLE.

Undernutrition in humans with IBD has been shown to significantly increase the duration of hospitalization.^21^ The cause of this finding is not fully understood, but is considered to be a result of the increased morbidity associated with undernutrition.^22^ A similar effect of undernutrition is likely to be responsible for the increased duration of hospitalization seen in dogs with higher USS in our study. Nutritional assessment at admission could help manage client expectations regarding duration of stay and the associated costs. Future studies are warranted to identify if addressing undernutrition has a positive impact on duration of hospitalization or overall outcome. However, because of factors unrelated to disease severity that might impact the duration of hospitalization (e.g., financial factors, demeanor of the patient), studies that take potential confounding factors into consideration are required.

Additional limitations of our study include that it was an observational study, and the treatment administered at diagnosis was not standardized. Significant variation was found in the combinations of different drugs used, and the dose of prednisolone administered, as well as in dietary management chosen after the diagnosis of PLE. The daily caloric intake for each dog during and after hospitalization also was not available, and therefore the effect of inadequate intake after diagnosis on outcome could not be established. A standardized treatment regimen including dietary management and caloric intake assessment would be required in future studies to minimize the effect of various drugs and diet types, as well as to determine the effects of calories consumed on outcome. Our study included 1 dog that was receiving treatment for osteoarthritis and 4 that were receiving glucocorticoids at the time of diagnosis, which may have impacted muscle condition. However, the inclusion of dogs receiving glucocorticoids was in agreement with studies assessing undernutrition scores in humans with IBD.^2^ Finally, because every section of the intestine was not biopsied in every dog, occult neoplasia as the cause of the PLE could not be excluded.

## 5 CONCLUSION

In conclusion, in dogs with PLE, a higher USS is associated with increased duration of hospitalization, failure to respond to initial treatment (prescribed at diagnosis), and failure to achieve clinical remission within 6 months. Of the 5 variables assessed in the USS, the combination of epaxial muscle loss and coat condition was most predictive of death and not achieving clinical remission within 6 months in dogs with PLE. However, additional prospective studies including treatment groups with defined treatment protocols detailing formulation and dosages of medication administered, which address some limitations of our study, need to be performed. These studies also would be required to determine if a change in USS and the associated 5 variables after diagnosis impacts outcome variables in these dogs.

## CONFLICT OF INTEREST DECLARATION

Authors declare no conflict of interest.

## OFF‐LABEL ANTIMICROBIAL DECLARATION

Authors declare no off‐label use of antimicrobials.

## INSTITUTIONAL ANIMAL CARE AND USE COMMITTEE (IACUC) OR OTHER APPROVAL DECLARATION

Approved by the Royal Veterinary College Clinical Research and Ethical Review Board (reference URN 2019 1861‐3).

## HUMAN ETHICS APPROVAL DECLARATION

Authors declare human ethics approval was not needed for this study.

## Supporting information


**Data S1.** Supporting Information
