# Supplementary material for: An undernutrition screening score for dogs with protein‐losing enteropathy: A prospective multicenter study
Source: J Vet Intern Med. 2023 Jul 21;37(5):1821–9. doi: 10.1111/jvim.16794 (PMC10472980; doi:10.1111/jvim.16794)
Supplement: Supplementary file 1 — Data S1. Supporting Information [file JVIM-37-1821-s001.pdf]

Biochemical data and intestinal histopathology results

The mean albumin concentration at presentation was 1.8 g/dL (SD ±0.5 g/dL; reference interval 2.8 – 3.8 g/dL). Twenty-three dogs (40%) had serum vitamin B12 concentrations lower than the reference interval and the remaining 34 dogs (60%) had values within the reference interval. The mean blood urea nitrogen concentration was 4.89 mmol/l (SD ±2.15; reference interval 3.1 – 10.1mmol/L). The mean albumin concentration at presentation was 1.8 g/dL (SD ±0.5 g/dL; reference interval 2.8 – 3.8 g/dL). Basal cortisol or ACTH stimulation test ruled out hypoadrenocorticism in 51 cases. The remaining six cases had either recently received glucocorticoids (n=5) or had evidence of a stress leucogram on complete blood count (n=1).

Urinalysis was performed in 53 dogs, with urine protein:creatinine ratio performed if urine dipstick documented proteinuria. The four that did not have a urinalysis performed had no evidence of PLN based on the presence of low (n=2) or low normal (n=2) globulin and cholesterol concentrations, alongside findings consistent with a chronic inflammatory enteropathy on intestinal biopsy specimens (n=4). Canine pancreatic lipase (cPLI) was performed in 16 cases, and was within the laboratory reference interval for 13 dogs (<200 ug/L), with 3 dogs having increased concentrations, median 880 ug/L (range 593 – 1459 ug/L). Fecal culture was performed in 19 dogs and was negative for all cases. Trypsin like immunoreactivity results excluded exocrine pancreatic insufficiency in all dogs for which it was assessed (n=35; TLI > 6 ug/L).

All dogs included in the study had intestinal biopsies performed. The majority of cases (56/57) had endoscopy performed in order to obtain intestinal mucosal pinch biopsies. Of these 56 dogs, 55 had an upper GI endoscopy and 41 had concurrent lower GI endoscopy in which the ileum was successfully intubated in 32 cases. One dog had only a lower GI endoscopy in which the ileum was successfully intubated. One dog underwent an exploratory laparotomy to obtain jejunal full thickness biopsies. All dogs were diagnosed with chronic inflammatory enteropathy based on histopathologic assessment of biopsies. In addition, small intestinal lacteal dilation was identified in 30, lymphangiectasia in 12 and crypt abscesses in 17 cases.

The small intestinal biopsy specimens obtained from the upper GI endoscopy and exploratory laparotomy identified: lymphoplasmacytic enteritis in 25 (45%) cases, lymphoplasmacytic and eosinophilic enteritis in 6 (11%), lymphoplasmacytic, eosinophilic and neutrophilic enteritis in 7 (12%), lymphoplasmacytic and neutrophilic enteritis in 10 (18%), plasmacytic enteritis in 7 (12%) and lymphocytic enteritis in 1 (2%).

The inflammation in ileal biopsies was characterized as: lymphoplasmacytic in 11 (35%) cases, lymphoplasmacytic and eosinophilic in 8 (25%), lymphoplasmacytic, eosinophilic and neutrophilic in 2 (6%), lymphoplasmacytic and neutrophilic in 7 (22%), and 1 each (3%) of the following: plasmacytic, plasmacytic and, lymphocytic, and plasmacytic and neutrophilic.

Colonic biopsies obtained by lower GI endoscopy identified: lymphoplasmacytic colitis in 12 (29%) cases, lymphoplasmacytic and eosinophilic colitis in 5 (12%), lymphoplasmacytic, eosinophilic and neutrophilic colitis in 5 (12%), lymphoplasmacytic and neutrophilic colitis in 4 (10%), plasmacytic colitis in 3 (7%), granulomatous colitis in 3 (7%), plasmacytic and eosinophilic colitis in 1 (2%), fibrosis in 3 (7%) and no significant abnormalities in 6 (14%).

Additional treatment of PLE at diagnosis:

A variety of antibiotics were administered including metronidazole (n=1), enrofloxacin (n=3), amoxicillin clavulanic acid (n=2), oxytetracycline (n=1) and clarithromycin (n=1). Prednisolone was administered to 41 dogs with a median dose of 2 mg/kg/day (range 0.5-2.2). Vitamin B12 was supplemented in all cases with low serum vitamin B12 concentrations (n=23) and in twelve cases with a serum vitamin B12 within the reference interval (range 201 – 308 ng/L). Vitamin B12 was supplemented orally in 24, subcutaneously in five and subcutaneously followed by orally in six dogs. Clopidogrel was administered to eight dogs and aspirin to five dogs. A variety of supportive GI medications were administered including: omeprazole (n=5), mirtazapine (n=3), ondansetron (n=2), maropitant (n=2), metoclopramide (n=1) and cisapride (n=1).
